# Supplementary material for: Selection Behavior of the Beet Armyworm, Spodoptera exigua (Hübner) Between Bt Maize and Conventional Maize Plants
Source: Insects. 2025 Oct 17;16(10):1059. doi: 10.3390/insects16101059 (PMC12564333; doi:10.3390/insects16101059)
Supplement: Supplementary file 1 [file insects-16-01059-s001.zip › insects-3891031-supplementary.pdf]

Supporting information

Table S1 Model fitting statistics for the relationship between larval feeding preference and feeding time under continuous exposure to single-type maize leaves (Bt or non-Bt).

Refuge proportion	Model	Non-Bt maize					Bt maize				
		P-value	*F*-value	R^2	AIC	BIC	*P*-value	*F*-value	R^2	AIC	BIC
1st instar	Logarithmic	0.000	41.606	0.839			0.801	0.068	0.008		
	Inverse	0.060	4.790	0.375			0.947	0.005	0.001		
	Quadratic	0.000	97.426	0.965	55.020	66.230	0.014	8.339	0.704	66.089	67.300
	Cubic	0.000	56.581	0.966	56.864	58.376	0.047	4.917	0.711	67.867	69.380
	Compound	0.000	39.013	0.830			0.184	2.113	0.209		
	Power	0.000	45.226	0.850			0.581	0.331	0.040		
	S-curve	0.047	5.521	0.408			0.803	0.067	0.008		
	Growth	0.000	39.013	0.830			0.184	2.113	0.209		
	Exponential	0.000	39.013	0.83			0.184	2.113	0.209		
	Logistic	0.000	39.013	0.83			0.184	2.113	0.209		
2nd instar	Logarithmic	0.663	0.205	0.025			0.002	22.155	0.735		
	Inverse	0.821	0.054	0.007			0.005	14.68	0.647		
	Quadratic	0.001	23.25	0.869	45.386	46.597	0.043	5.118	0.594	75.679	76.889
	Cubic	0.004	13.701	0.873	47.118	48.631	0.034	5.710	0.741	73.196	74.709
	Compound	0.115	3.119	0.280			0.020	8.303	0.509		
	Power	0.626	0.257	0.031			0.002	19.684	0.711		
	S-curve	0.856	0.035	0.004			0.011	10.676	0.572		
	Growth	0.115	3.119	0.280			0.020	8.303	0.509		
	Exponential	0.115	3.119	0.28			0.020	8.303	0.509		
3rd instar	Logarithmic	0.352	0.978	0.109			0.046	5.591	0.411		
	Inverse	0.298	1.237	0.134			0.001	22.537	0.738		
	Quadratic	0.036	5.569	0.614	62.836	64.046	0.076	3.814	0.521	74.070	75.280
	Cubic	0.102	3.258	0.620	64.691	66.204	0.031	5.941	0.748	69.651	71.164
	Compound	0.828	0.051	0.006			0.572	0.347	0.042		
	Power	0.339	1.033	0.114			0.071	4.315	0.350		
	S-curve	0.292	1.271	0.137			0.005	14.847	0.650		
	Growth	0.828	0.051	0.006			0.572	0.347	0.042		
	Exponential	0.828	0.051	0.006			0.572	0.347	0.042		
	Logistic	0.828	0.051	0.006			0.572	0.347	0.042		
4th instar	Logarithmic	0.094	3.598	0.310			0.027	7.364	0.479		
	Inverse	0.245	1.575	0.164			0.098	3.509	0.305		
	Quadratic	0.102	3.221	0.479	63.861	65.071	0.147	2.547	0.421	67.155	68.365
	Cubic	0.139	2.698	0.574	63.845	65.358	0.080	3.731	0.651	64.096	65.609
	Compound	0.034	6.513	0.449			0.052	5.199	0.394		
	Power	0.104	3.369	0.296			0.029	7.031	0.468		
	S-curve	0.250	1.541	0.162			0.104	3.370	0.296		
	Growth	0.034	6.513	0.449			0.052	5.199	0.394		
	Exponential	0.034	6.513	0.449			0.052	5.199	0.394		
	Logistic	0.034	6.513	0.449			0.052	5.199	0.394		
5th instar	Logarithmic	0.010	11.191	0.583			0.066	4.512	0.361		
	Inverse	0.063	4.666	0.368			0.060	4.793	0.375		
	Quadratic	0.037	5.463	0.609	50.549	51.759	0.068	4.035	0.535	64.123	62.333
	Cubic	0.072	3.931	0.663	51.082	52.595	0.045	5.003	0.714	58.258	59.771

	Compound	0.051	5.253	0.396			0.395	0.808	0.092		
	Power	0.011	10.873	0.576			0.075	4.173	0.343		
	S-curve	0.065	4.577	0.364			0.066	4.534	0.362		
	Growth	0.051	5.253	0.396			0.395	0.808	0.092		
	Exponential	0.051	5.253	0.396			0.395	0.808	0.092		
	Logistic	0.051	5.253	0.396			0.395	0.808	0.092		

Note: This table presents the results of several nonlinear regression models fitted to describe the temporal dynamics of larval feeding preference under continuous feeding conditions. For each model and larval instar, the corresponding *P*-value, *F*-value, and coefficient of determination (R^2) are reported. Polynomial models (quadratic and cubic) were selected for further analysis based on the proportion of variance explained (highest and second-highest R^2 values). Generalized linear models were used to compute Akaike's Information Criterion (AIC) and Bayesian Information Criterion (BIC) for model comparison. Both quadratic and cubic models provided comparable AIC values; however, cubic models generally exhibited higher R^2 and accounted for the majority of best-fitting cases. Therefore, cubic functions were retained for subsequent analyses of feeding preference. Blank entries in the AIC and BIC columns indicate that these values were not calculated for the corresponding models.

Table S2 Model fitting statistics for the relationship between larval feeding preference and feeding time in the continuous feeding choice test with alternating Bt and non-Bt leaves.

Refuge proportion	Model	Non-Bt maize					Bt maize				
		P-value	*F*-value	R^2	AIC	BIC	*P*-value	*F*-value	R^2	AIC	BIC
1st instar	Logarithmic	0.001	28.114	0.778			0.057	4.946	0.382		
	Inverse	0.086	3.821	0.323			0.016	9.199	0.535		
	Quadratic	0.000	34.075	0.907	56.065	57.275	0.445	0.910	0.206	66.290	67.500
	Cubic	0.001	20.497	0.911	57.598	59.111	0.184	2.238	0.528	63.091	64.603
	Compound	0.000	34.202	0.810			0.177	2.196	0.215		
	Power	0.001	27.409	0.774			0.080	4.024	0.335		
	S-curve	0.079	4.036	0.335			0.039	6.074	0.432		
	Growth	0.000	34.202	0.810			0.177	2.196	0.215		
	Exponential	0.000	34.202	0.810			0.177	2.196	0.215		
	Logistic	0.000	34.202	0.810			0.177	2.196	0.215		
2nd instar	Logarithmic	0.024	7.765	0.493			0.004	15.827	0.664		
	Inverse	0.356	0.959	0.107			0.039	6.065	0.431		
	Quadratic	0.004	13.874	0.799	67.103	68.313	0.005	12.003	0.774	55.911	57.121
	Cubic	0.003	15.652	0.887	63.348	64.861	0.023	6.864	0.774	57.905	59.418
	Compound	0.001	22.339	0.736			0.025	7.585	0.487		
	Power	0.037	6.276	0.440			0.007	13.277	0.624		
	S-curve	0.417	0.732	0.084			0.057	4.945	0.382		
	Growth	0.001	22.339	0.736			0.025	7.585	0.487		
	Exponential	0.001	22.339	0.736			0.025	7.585	0.487		
3rd instar	Logarithmic	0.597	0.304	0.037			0.221	1.760	0.180		
	Inverse	0.566	0.359	0.043			0.016	9.317	0.538		
	Quadratic	0.287	1.498	0.300	42.644	43.854	0.296	1.457	0.294	65.977	67.187
	Cubic	0.133	2.773	0.581	39.509	41.022	0.007	11.539	0.852	52.334	53.847
	Compound	0.479	0.552	0.065			0.877	0.026	0.003		
	Power	0.63	0.251	0.030			0.301	1.223	0.133		
	S-curve	0.577	0.339	0.041			0.030	6.992	0.466		

	Growth	0.479	0.552	0.065			0.877	0.026	0.003		
	Exponential	0.479	0.552	0.065			0.877	0.026	0.003		
	Logistic	0.479	0.552	0.065			0.877	0.026	0.003		
4th instar	Logarithmic	0.284	1.319	0.142			0.809	0.063	0.008		
	Inverse	0.254	1.511	0.159			0.233	1.663	0.172		
	Quadratic	0.198	2.056	0.370	69.671	70.881	0.927	0.076	0.021	54.589	55.799
	Cubic	0.245	1.814	0.476	69.836	71.349	0.191	2.182	0.522	49.428	50.940
	Compound	0.694	0.166	0.020			0.725	0.132	0.016		
	Power	0.263	1.452	0.154			0.797	0.07	0.009		
	S-curve	0.253	1.520	0.160			0.226	1.724	0.177		
	Growth	0.694	0.166	0.020			0.725	0.132	0.016		
	Exponential	0.694	0.166	0.020			0.725	0.132	0.016		
	Logistic	0.694	0.166	0.020			0.725	0.132	0.016		
5th instar	Logarithmic	0.142	2.659	0.249			0.014	9.840	0.552		
	Inverse	0.296	1.249	0.135			0.019	8.492	0.515		
	Quadratic	0.177	2.243	0.391	60.656	61.866	0.094	3.373	0.491	68.923	71.033
	Cubic	0.321	1.442	0.419	62.177	63.690	0.164	2.421	0.548	70.640	72.153
	Compound	0.062	4.701	0.370			0.061	4.754	0.373		
	Power	0.142	2.650	0.249			0.016	9.347	0.539		
	S-curve	0.286	1.307	0.140			0.027	7.262	0.476		
	Growth	0.062	4.701	0.370			0.061	4.754	0.373		
	Exponential	0.062	4.701	0.370			0.061	4.754	0.373		
	Logistic	0.062	4.701	0.370			0.061	4.754	0.373		

Note: This table presents the results of several nonlinear regression models fitted to describe the temporal dynamics of larval feeding preference under continuous feeding conditions. For each model and larval instar, the corresponding *P*-value, *F*-value, and coefficient of determination (R^2) are reported. Polynomial models (quadratic and cubic) were selected for further analysis based on the proportion of variance explained (highest and second-highest R^2 values). Generalized linear models were used to compute Akaike's Information Criterion (AIC) and Bayesian Information Criterion (BIC) for model comparison. Both quadratic and cubic models provided comparable AIC values; however, cubic models generally exhibited higher R^2 and accounted for the majority of best-fitting cases. Therefore, cubic functions were retained for subsequent analyses of feeding preference. Blank entries in the AIC and BIC columns indicate that these values were not calculated for the corresponding models.

Table S3 Model fitting statistics for the relationship between survey day and larval mean and maximum dispersal distances under seed-mixture refuge patterns.

Refuge proportion	Model	Mean dispersal distances					Maximum dispersal distances				
		P-value	*F*-value	R^2	AIC	BIC	*P*-value	*F*-value	R^2	AIC	BIC
0%	Logarithmic	0.522	0.445	0.047			0.368	0.897	0.091		
	Inverse	0.924	0.010	0.001			0.930	0.008	0.001		
	Quadratic	0.093	3.248	0.448	50.936	52.527	0.089	3.331	0.454	97.995	99.586
	Cubic	0.032	5.319	0.695	46.410	48.400	0.027	5.707	0.710	93.050	95.039
	Compound	—	—	—			—	—	—		
	Power	—	—	—			—	—	—		
	S-curve	—	—	—			—	—	—		
	Growth	—	—	—			—	—	—		

	Exponential	—	—	—			—	—	—		
	Logistic	—	—	—			—	—	—		
5%	Logarithmic	0.127	2.835	0.240			0.028	6.866	0.433		
	Inverse	0.556	0.374	0.040			0.242	1.568	0.148		
	Quadratic	0.066	3.889	0.493	53.880	55.472	0.008	9.518	0.704	89.504	91.096
	Cubic	0.002	15.325	0.868	41.089	53.089	0.000	59.068	0.962	68.928	70.917
	Compound	—	—	—			—	—	—		
	Power	—	—	—			—	—	—		
	S-curve	—	—	—			—	—	—		
	Growth	—	—	—			—	—	—		
	Exponential	—	—	—			—	—	—		
10%	Logarithmic	0.455	0.609	0.063			0.995	0.0000	0.000		
	Inverse	0.169	2.237	0.199			0.589	0.313	0.034		
	Quadratic	0.058	4.134	0.508	52.940	54.930	0.079	3.535	0.469	100.740	102.331
	Cubic	0.050	4.346	0.651	54.700	56.292	0.071	3.662	0.611	99.325	101.314
	Compound	0.313	1.140	0.112			0.418	0.720	0.074		
	Power	0.054	4.922	0.354			0.939	0.006	0.001		
	S-curve	0.002	18.231	0.669			0.579	0.332	0.036		
	Growth	0.313	1.140	0.112			0.418	0.720	0.074		
	Exponential	0.313	1.140	0.112			0.418	0.720	0.074		
	Logistic	0.313	1.140	0.112			0.418	0.720	0.074		
20%	Logarithmic	0.000	54.834	0.859			0.000	46.575	0.838		
	Inverse	0.003	16.491	0.647			0.000	92.352	0.911		
	Quadratic	0.000	39.290	0.908	56.510	58.101	0.001	17.267	0.812	85.795	87.387
	Cubic	0.000	30.470	0.929	55.632	57.622	0.001	17.740	0.884	82.502	84.491
	Compound	—	—	—			—	—	—		
	Power	—	—	—			—	—	—		
	S-curve	—	—	—			—	—	—		
	Growth	—	—	—			—	—	—		
	Exponential	—	—	—			—	—	—		
	Logistic	—	—	—			—	—	—		
100%	Logarithmic	0.000	62.941	0.875			0.000	46.195	0.837		
	Inverse	0.002	18.381	0.671			0.000	38.800	0.812		
	Quadratic	0.000	44.521	0.918	64.126	65.718	0.000	27.801	0.874	82.022	83.614
	Cubic	0.000	29.914	0.928	64.692	66.681	0.001	21.668	0.903	81.188	83.178
	Compound	0.001	24.714	0.733			0.006	12.757	0.586		
	Power	0.000	92.073	0.911			0.000	34.929	0.795		
	S-curve	0.000	58.225	0.866			0.000	37.357	0.806		
	Growth	0.001	24.714	0.733			0.006	12.757	0.586		
	Exponential	0.001	24.714	0.733			0.006	12.757	0.586		
	Logistic	0.001	24.714	0.733			0.006	12.757	0.586		

Note: Several nonlinear regression models were fitted to describe the temporal dynamics of larval dispersal. For each model, the corresponding *P*-value, *F*-value, and coefficient of determination (R^2) are reported. Based on the proportion of variance explained (i.e., the highest and second-highest R^2 values), polynomial models (quadratic and cubic) were selected for further analysis. Generalized linear models were then applied to compute Akaike's Information Criterion (AIC) and Bayesian Information Criterion (BIC) for model comparison. The cubic polynomial model consistently yielded the lowest AIC values and highest R^2, indicating superior statistical performance. However, visual inspection of the fitted curves

revealed biologically unrealistic negative values for dispersal distance at later time points. To ensure biological plausibility, we therefore selected the quadratic polynomial model, which provided an adequate fit (with relatively high R^2 and low AIC) while avoiding implausible predictions. Blank entries in the AIC and BIC columns indicate that these values were not calculated for the corresponding model.
